# Diffuse Adenomatosis and Hepatocellular Carcinoma Treated with Liver Transplantation in an Adolescent Female with Kabuki Syndrome with a Novel *KMT2D* Gene Mutation

**DOI:** 10.1155/2019/7983824

**Published:** 2019-04-24

**Authors:** Leander D. Timothy, Heidi D. Lehrke, Vishal S. Chandan, Amy B. Kolbe, Katryn N. Furuya

**Affiliations:** ^1^Department of Pediatric and Adolescent Medicine, Mayo Clinic, 200 First St. SW, Rochester, MN 55905, USA; ^2^Department of Laboratory Medicine and Pathology, Mayo Clinic, 200 First St. SW, Rochester, MN 55905, USA; ^3^Division of Anatomic Pathology, Mayo Clinic, 200 First St. SW, Rochester, MN 55905, USA; ^4^Department of Radiology, Mayo Clinic, 200 First St. SW, Rochester, MN 55905, USA; ^5^Division of Pediatric Radiology, Mayo Clinic, 200 First St. SW, Rochester, MN 55905, USA; ^6^Division of Pediatric Gastroenterology and Hepatology, Mayo Clinic, 200 First St. SW, Rochester, MN 55905, USA

## Abstract

Kabuki syndrome (KS) is a rare disorder primarily associated with mutations in the *KMT2D* and *KDM6A* genes. Several tumors have been reported with KS; however, there have been no reports of hepatocellular carcinoma (HCC) or hepatic adenomatosis. We present an adolescent girl with KS and a novel *KMT2D* mutation who developed diffuse adenomatosis, HCC, and subsequently underwent liver transplantation.

## 1. Introduction

Kabuki syndrome (KS) is a rare disorder (1 : 32000 births) characterized by distinctive facial features including eversion of the lower lateral eyelids, arched eyebrows, depressed nasal tip, and prominent ears; skeletal abnormalities, such as brachydactyly and spinal and digital anomalies; mild to moderate cognitive delay; and postnatal growth impairment [1].

Mutations in *KMT2D* (MIM #602113) at 12q13.12 account for 55–80% of patients, while 9–14% of *KMT2D*-negative patients have deletions or mutations in the *KDM6A* (MIM #300128) gene at Xp11.3 [2]. However, absence of these or other chromosomal abnormalities does not preclude a diagnosis of KS if the clinical phenotype is present [3].

Increasing numbers of tumors have been reported concurrently in KS including hepatoblastoma, neuroblastoma, pilomatricoma, pre-B-cell acute lymphoblastic leukemia, spinal ependymoma, and synovial sarcoma [4–7]; however, hepatocellular carcinoma (HCC) and adenomatosis have not previously been reported.

HCC is the second most common pediatric liver tumor, accounting for 87% of liver tumors among adolescents [8]. Hepatic adenomatosis (HA) is characterized by multiple adenomas in an otherwise normal liver [9]. While considered a benign disease, some patients may develop potential fatal complications, with malignant transformation to HCC occurring in less than 10% of cases [9].

We describe a novel case of KS due to a heterozygous mutation in exon 31 of the *KMT2D* gene, c.8594dupC, who presented with HA and HCC.

## 2. Clinical Report

A 15-year-old female with KS diagnosed at 5 years of age presented with an incidental hepatic mass found during a follow-up echocardiogram of her repaired atrial septal defect. She was referred to Liver Clinic with complaints of intermittent abdominal pain, occasional dark urine in the preceding two months, and unexplained pruritus for 10 years. There was no history of jaundice, change in stool color, hematochezia, melena, fevers, anorexia, or recent weight loss. Her past medical history included medullary nephrocalcinosis, intermittent microhematuria, IgG deficiency, asthma, bilateral conductive hearing loss, and developmental delay. She had also been started on a low-dose estrogen containing oral contraceptive pill (OCP) 1 year prior to presentation which had been increased to a high-dose estrogen containing OCP six months previously for menorrhagia. The only other medications she had been on were Risperdal and a multivitamin. Physical examination revealed facial features consistent with KS as well as esotropia. She was noted to have palmar erythema but no other stigmata of chronic liver disease. She had hepatosplenomegaly with a firm palpable epigastric mass that extended 8 cm below the xiphoid process.

Biochemical analysis demonstrated the following: AST 65 U/L, ALT 50 U/L, GGT 85 U/L, total bilirubin 1.2 mg/dL, direct bilirubin 0.6 mg/dL with normal complete blood count, electrolytes, urea, and creatinine. Alpha-fetoprotein and CA-19-9 were not elevated. Nonfasting bile acid profiles were obtained at her initial visit and revealed elevated cholic acid, chenodeoxycholic acid, and total bile acids. Tests for Wilson disease, alpha-1-antitrypsin, viral hepatitis, and autoimmune hepatitis were all negative.

**Figure 1:**
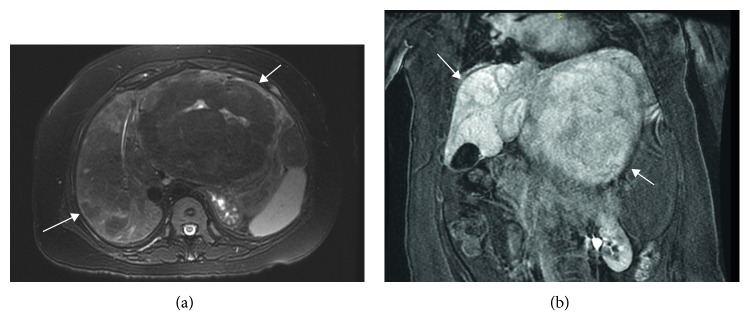
(a) Axial T2 with fat saturation: large predominantly T2 hypointense mass in left hepatic lobe, T2 hyperintense scar internally, and heterogeneous liver parenchyma in the right lobe with suggestion of additional T2 hypointense masses. (b) Coronal postgadolinium LAVA: 19 min delay. Many of the masses retain hepatobiliary agent (Eovist) relative to the background liver on delayed series which suggests presence of functioning hepatocytes, typically seen in focal nodular hyperplasia, but can be seen in inflammatory subtype of adenomas and even well-differentiated HCC. Arrows designate multiple masses.

Imaging included an abdominal CT scan that demonstrated a 15 cm diameter left hepatic lobe mass with heterogeneous enhancement and coarse calcifications. Similar findings were present on abdominal MRI performed with Gadavist and Eovist. A 15 × 11 × 14.5 cm heterogeneous mass within the left hepatic lobe with retention of hepatobiliary contrast was demonstrated on abnormal background liver parenchyma with multiple other similar appearing smaller masses in both hepatic lobes (Figures 1(a) and 1(b)). These lesions were thought to be hepatic adenomas, as the inflammatory subtype is known to sometimes retain hepatobiliary contrast [10]. Occlusion of the main portal vein was noted, and splenomegaly was present.

An ultrasound-guided liver biopsy taken of the large left hepatic lobe lesion histologically demonstrated an atypical hepatic adenoma with possible early malignant transformation. Reticulin staining showed focal disruption of the reticulin meshwork with thickened liver plates and a slightly increased Ki-67 proliferation index. Additionally, biopsy of the background hepatic parenchyma in the right hepatic lobe demonstrated benign appearing hepatocytes with changes suggestive of blood flow abnormalities. There was no evidence to suggest the presence of glycogen storage disease.

**Figure 2:**
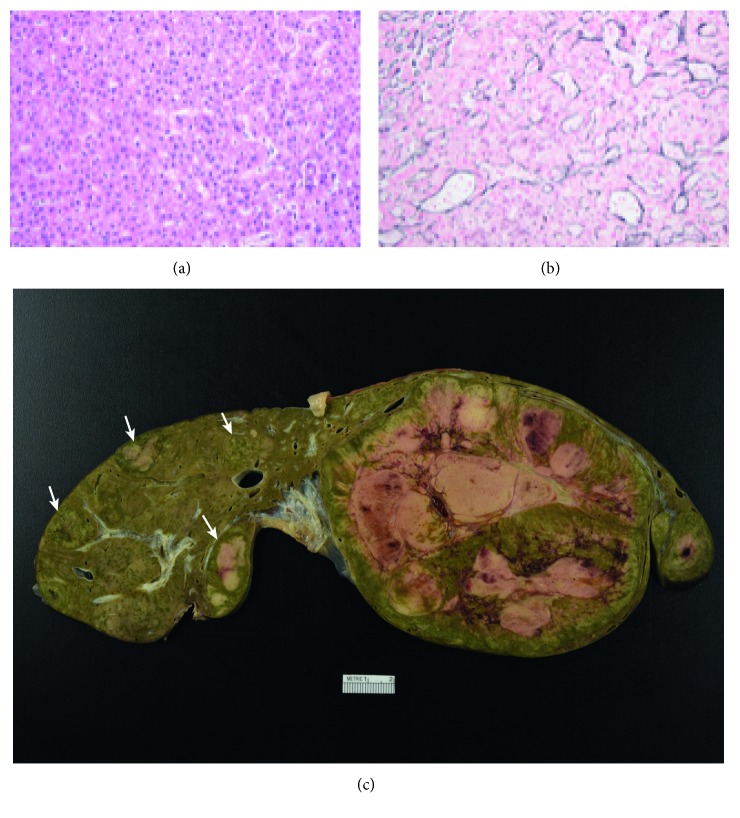
(a) Photomicrograph of the large hepatic mass reveals a well-differentiated hepatocellular neoplasm with mild cytologic atypia. A single unpaired vessel is present at the right-hand side of the image (hematoxylin and eosin, 20x magnification). (b) The large hepatic mass shows a focally disrupted reticulin meshwork with thickened hepatocyte trabeculae, consistent with a well-differentiated hepatocellular carcinoma (reticulin stain, 20x magnification). (c) The explanted specimen reveals a large heterogeneous mass with areas of hemorrhage and necrosis, occupying nearly the entire left hepatic lobe (hepatocellular carcinoma arising from a hepatic adenoma). Multiple small hepatic adenomas are present within the right hepatic lobe (arrows).

She was subsequently listed and underwent deceased donor liver transplantation. The explanted liver demonstrated numerous hepatic adenomas. Within the left hepatic lobe adenoma (19.5 × 9.8 × 9.5 cm) (Figure 2(c)), hepatocytes with mild cytologic atypia were found in areas of reticulin disruption, consistent with well-differentiated HCC arising from a hepatic adenoma with areas of macrovesicular steatosis and scattered foci of extramedullary hematopoiesis. There were multiple satellite lesions (>10) which were also well-differentiated hepatocellular neoplasms and most consistent with hepatic adenomas. This was supported by a reticulin stain demonstrating an intact reticulin meshwork, absent glypican-3 staining, and a low Ki-67 proliferation index (Figures 2(a) and 2(b)).

## 3. Discussion

Liver tumors occur rarely in children. HCC usually develops in the context of underlying liver disease that gives rise to cirrhosis such as hepatitis B, hepatitis C, nonalcoholic steatohepatitis, or metabolic/genetic disease such as glycogen storage disease or tyrosinemia. Genetic diseases may also lead to the development of hepatocarcinogenesis, and in particular, those that alter tumor suppression pathways [8]. Our patient developed hepatocellular carcinoma on a background of hepatic adenomatosis. She had been on an OCP for 1 year prior to the diagnosis of HCC. This certainly may have contributed to the development of these tumors, given her underlying genetic predisposition to the development of malignancy. While corticosteroid use and underlying conditions such as glycogen storage disease have been cited as risk factors for the development of hepatic adenomas, this was not the case in our patient.

*KMT2D* is part of the lysine methyltransferase superfamily that is a group of evolutionarily conserved transcriptional regulators that play an important role in metabolic processes as well as the regulation of development, differentiation, and tumor suppression [11]. *KMT2D* has been found to be mutated in HCC and has also been reported to be disrupted as a recurrent site of hepatitis B virus integration [12]. Furthermore, tumors with *KMT2D* family mutations may be associated with a trend toward earlier recurrence of disease, greater microvascular invasion, and a more aggressive phenotype [12]. Somatic mutations in *KMT2D* may also play an important role in other tumors including medulloblastoma [13], follicular lymphoma, diffuse large B-cell lymphoma [14], and prostate cancer [15]. It is postulated that mutations in the *KMT2D* gene may result in abnormal enhancer regulation and altered cell-type specific gene expression or may lead to changes in transcription, DNA breaks, and tumor formation [11].

*KMT2D* has also been found to be important in the regulation of the hepatic circadian clock and acts as a transcriptional coactivator of PPARƴ2 [16], and the normal rhythmic fluctuation of bile acid levels is eliminated in *KMT2D* mutant mice [17]. Elevated serum bile acids have been linked in a farnesoid X receptor mouse knock out model with increased expression of the Myc oncogene and hepatic tumor development [18]. Of note, our patient did have elevated serum bile acids; however, they were obtained in a nonfasting state.

## 4. Conclusion

This is the first report of HA and HCC occurring in a patient with Kabuki syndrome. There is growing concern that KS may predispose to the development of malignancy. Research into the possible role that *KMT2D* plays in tumorigenesis would further elucidate whether patients with KS should undergo routine cancer screening.

## Abbreviations

KS: Kabuki syndrome
HCC: Hepatocellular carcinoma
CT: Computed tomography
H3K4: Histone H3 lysine 4
ALT: Alanine aminotransferase
AST: Aspartate aminotransferase
GGT: Gamma-glutamyltransferase.
